# Multi-Dimensional Gene Regulation in Innate and Adaptive Lymphocytes: A View From Regulomes

**DOI:** 10.3389/fimmu.2021.655590

**Published:** 2021-03-25

**Authors:** Nilisha Fernando, Giuseppe Sciumè, John J. O’Shea, Han-Yu Shih

**Affiliations:** ^1^ Neuro-Immune Regulome Unit, National Eye Institute, National Institutes of Health, Bethesda, MD, United States; ^2^ Laboratory Affiliated to Istituto Pasteur Italia – Fondazione Cenci-Bolognetti, Department of Molecular Medicine, Sapienza University of Rome, Rome, Italy; ^3^ Lymphocyte Cell Biology Section, Molecular Immunology and Inflammation Branch, National Institute of Arthritis, Musculoskeletal and Skin Diseases, National Institutes of Health, Bethesda, MD, United States; ^4^ National Institute of Neurological Disorders and Stroke, National Institutes of Health, Bethesda, MD, United States

**Keywords:** signal-regulated transcription factors, lineage-determining transcription factors, *de novo* enhancers, poised enhancers, ATAC-seq and chromatin accessibility, innate lymphoid cell (ILC), histone modifications

## Abstract

The precise control of cytokine production by innate lymphoid cells (ILCs) and their T cell adaptive system counterparts is critical to mounting a proper host defense immune response without inducing collateral damage and autoimmunity. Unlike T cells that differentiate into functionally divergent subsets upon antigen recognition, ILCs are developmentally programmed to rapidly respond to environmental signals in a polarized manner, without the need of T cell receptor (TCR) signaling. The specification of cytokine production relies on dynamic regulation of cis-regulatory elements that involve multi-dimensional epigenetic mechanisms, including DNA methylation, transcription factor binding, histone modification and DNA-DNA interactions that form chromatin loops. How these different layers of gene regulation coordinate with each other to fine tune cytokine production, and whether ILCs and their T cell analogs utilize the same regulatory strategy, remain largely unknown. Herein, we review the molecular mechanisms that underlie cell identity and functionality of helper T cells and ILCs, focusing on networks of transcription factors and cis-regulatory elements. We discuss how higher-order chromatin architecture orchestrates these components to construct lineage- and state-specific regulomes that support ordered immunoregulation.

## Regulomes Define Divergent Lymphocyte Transcriptional Programs

Each nucleus contains six billion nucleotides compacted into nucleosomes as basic units of chromatin that are orderly compacted and compartmentalized for precise gene regulation (1, 2). Residing among 98% of non-coding mammalian genomes are nearly three million regulatory elements (REs) that control the expression of approximately 20,000 genes in a cell-specific manner upon developmental and environmental cues (3). In lymphocytes, large networks of REs and transcription factors (TFs) orchestrate transcriptional and phenotypic diversity (4–6). The majority of REs are enhancers that remotely modulate transcription from a distance. However, the mechanisms of how intrinsic and extrinsic cues control enhancer activities to coordinate cell type- and state-specific gene expression profiles are yet to be understood.

Innate lymphoid cells (ILCs) play critical roles in tissue homeostasis, barrier integrity and primary host defense and mirror the functionalities of their effector counterparts in the adaptive immune compartment, CD4^+^ helper T (Th) and CD8^+^ cytotoxic T lymphocytes (CTL) (7–10). The similarities between innate and adaptive lymphocyte programming have dramatically accelerated our understanding of ILC regulation using the knowledge accumulated from studies of T cells (11–14). Other innate-like T cells, such as NKT cells, that mirror their functional T cell analogs also reveal similar lineage programming during development at both transcriptomic and epigenomic levels, which is beyond the scope of this review (15, 16). Here, we will focus on how cell identity and function are epigenetically imprinted during ILC maturation and how environmental signals activate or maintain ILC regulomes that define their transcriptomes.

## Regulomes of ILCs and Their T Cell Doppelgängers

Immune responses mounted against pathogens can be categorized into three main programs (17). Type 1 immunity is manifested by IFN-γ production in natural killer (NK) cells, CTLs, type 1 ILC (ILC1) and type 1 Th cells (Th1) to control intracellular pathogens. Type 2 immunity is characterized by interleukin (IL)-4, IL-5, IL-9 and IL-13 production from ILC2 and Th2 cells in defense against extracellular helminths. Finally, type 3 immunity is defined by the production of IL-17 and IL-22 in ILC3 and Th17/22 cells to constrain extracellular fungi and bacteria (10, 18–20).

These distinct, but sometimes overlapping, programs are specified by key lineage-determining transcription factors (LDTFs) that shape regulomes by acting as master regulators to control lymphocyte development and differentiation (21–23). EOMES, a T-box family TF, oversees initial NK cell development and CTL differentiation into effector and memory stages (24–31). T-bet, another T-box family TF encoded by *Tbx21*, also directs the type 1 immune response by coordinating with EOMES for CTL memory establishment and maintenance and enforcing NK cell maturation. T-bet expression is exclusively essential for both lineage specification and function in ILC1 and Th1 cells, as these lymphocytes do not express EOMES (32, 33). High level expression of GATA-binding protein 3 (GATA-3, encoded by the *Gata3* gene) plays a key role in ILC2 and Th2 cell differentiation and cytokine production (34–36). Finally, type 3 immunity is governed by RAR-related orphan receptor gamma, RORγt (encoded by the *Rorc* gene), which controls ILC3 and Th17 lineage specification and cytokine secretion (37, 38). These LDTFs epigenetically activate and stabilize function-related gene expression and, at the same time, inhibit transcription of genes that contribute to alternative cell fates (8, 39).

REs are typically characterized as conserved non-coding DNA sequences that become nucleosome-depleted to permit TF binding. For many years, identification and characterization of functional REs required extraordinary but often imprecise efforts. Use of computational prediction of REs through sequence conservation provided candidates that required further validation by assessment of chromatin accessibility by a DNase hypersensitivity assay or chromatin immunoprecipitation (ChIP) assays using antibodies directed at acetylated histone marks (40, 41). Similarly, the crosstalk between REs, such as enhancer-promoter interactions, has been measured by chromatin conformation capture (3C) or 3C-based assays (42). However, the development of massively parallel genomic DNA sequencing incorporating with conventional assays (e.g. DNase-seq, ChIP-seq, Hi-C) ushered in a new era of epigenomic research (43–46). These methods have been applied to map the regulomes of a wide range of immune cell populations, including T cells, B cells and macrophages (6, 47–52). CD4^+^ naïve T cells, for instance, establish lineage-specific regulomes during terminal differentiation that underlie Th cell identity and effector function (53–58). The improvement of relevant molecular biology techniques, including single cell RNA-seq (59), assay of transposase-accessible chromatin using sequencing (ATAC-seq) (60), ultra-low-input native ChIP-seq (61) and indexing first ChIP (iChIP) (62), further allows for the systematic interrogation of global transcriptomes and regulomes in low cell and rarer populations, including ILCs. Similar to their Th analogs, ILC subsets revealed cell-type restricted regulomes that define their lineage and effector competence (38, 63–66). These pre-programmed epigenomic configurations prime the REs at both TF and cytokine loci to maintain cell identity and enable rapid innate immune responses.

In contrast to Th cells that reshape naïve T-cell chromatin landscapes into divergent Th regulomes in response to combinational TCR and cytokine stimulation (54–56, 67), ILCs gradually construct lineage-specific, function-related regulomes during development prior to activation (38, 64). Un-supervised hierarchical clustering of murine immune cell regulomes clearly segregates ILCs from T lymphocytes (64). Similar results were obtained in humans when comparing type 1 and type 3 innate and adaptive lymphocytes from pediatric tonsils (63), consistent with the finding that regulomes are highly conserved across species (68).

Interestingly, while encountering challenges such as infection, innate and adaptive lymphocyte analogs converge their regulomes to execute overlapped effector activities to synergize host defense (64, 69). For example, upon *Nippostrongylus brasiliensis* infection in mice, naïve T cell regulomes are transformed into Th2 regulomes that resemble ILC2 regulomes, while ILC2 regulomes were minimally altered (64). Similarly, in mouse cytomegalovirus infection, effector NK cells and CD8^+^ T cells exhibit higher epigenomic commonality compared to naïve NK and CD8^+^ cells (69). Also, global DNA methylation patterns of adaptive NK cells in human cytomegalovirus were highly similar to the profile observed in CD8^+^ T cells (70). The convergence of ILC and T cell regulomes indicates a conservation of intrinsic regulatory networks in innate and adaptive compartments along with the impact of extrinsic signals.

**Figure 1:**
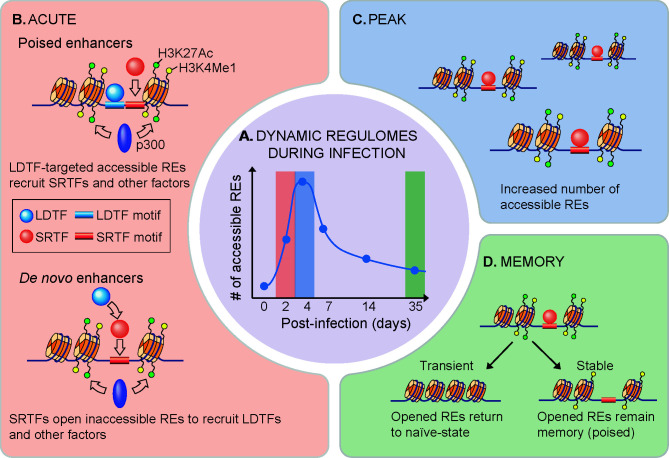
Dynamics of NK cell regulomes during infection. **(A)** Dynamic regulomes during infection. Innate immune response occurs along with changes in gene expression as well as chromatin accessibility. **(B)** High-magnitude gene upregulation during NK cell activation relies on recruitment of signal-regulated transcription factors (SRTFs) to poised enhancers that are developmentally acquired in a lineage-determining transcription factors (LDTFs) manner for chromatin remodeling (top) (72). High-magnitude gene induction also forms *de novo* enhancers through a process involving sequence-specific binding of SRTFs to inaccessible chromatin regions, followed by LDTF recruitment and enhancer activation (bottom) (72). **(C)** Formation of new accessible sites rapidly occurs *in vivo* upon mouse cytomegalovirus or *Toxoplasma gondii* infection until a peak of the response is reached (69). **(D)** At the end of viral infection, majority of these rapidly opened chromatins return to resting state, while part of them undergo stable epigenetic poising that maintains NK cell adaptive-like or memory phenotype (69, 70).

During the course of mouse cytomegalovirus infection, NK cells acquire an adaptive-like phenotype that provides memory responses similarly to those of T cells (71). This process involves acquisition of both stable and transient epigenetic changes, although the majority of accessible sites return to the naïve state (**Figure 1**) (69). Notably, naïve and memory CD8^+^ T cell regulomes are clustered in proximity in the un-supervised hierarchical clustering analysis, suggesting a naïve-like chromatin landscape in memory T cells (64). Upon NK cell activation, REs associated with *Socs3*, *Cish*, *Pdcd1*, *Dnmt3a*, and *Il10* gene loci acquire stable DNA-accessibility, while REs near *Tbx21*, *Klrg1*, *Ifng*, and *Zbtb32* are associated with transient modifications (69). Interferon-stimulated response element-like sequences were enriched in peaks remaining accessible over time, while motifs for TCF–LEF and NF-κB family members were enriched in regions becoming less accessible and undergoing epigenetic poising (69). Nevertheless, in contrast to naïve and infected ILC regulomes that are clustered in close proximity, terminally differentiated effector Th cells are clustered distally from naïve T cells (64). A recent study indicates that environmental challenges like microbes in gut heavily contribute to the continuous effector Th cell distribution of both transcriptomes and epigenomes (73). This finding suggests that adaptive T cells bear a more plastic character as compared to ILCs.

## Transcription Factors Shape Lymphocyte Subset Regulomes

During development, inaccessible REs are recognized by pioneer TFs in a sequence-specific manner. This is followed by chromatin remodeling, which propagates heritable epigenetic information that instructs cell identity (74). LDTFs are often considered as pioneer TFs, specifying lymphocyte lineage fates by targeting selective REs. In macrophages and B cells, PU.1 is an LDTF that occupies the majority of the active enhancers and is required for nucleosome remodeling and histone 3 lysine 4 methylation of these REs (75). The enrichment of T-bet, GATA-3 and RORγt motifs in type 1, 2 and 3 ILC-specific accessible chromatins, respectively, leads to the question whether LDTFs can directly open the chromatin or cooperate with other factors to shape ILC regulomes during development (23, 63, 64). More recently, the integration of transcriptomic analysis and TF motif analysis, obtained by chromatin accessibility data, has been applied to predict the role for almost one hundred TFs in the regulation of ILC identity, in mice. These data reveal the ability of TFs to both activate or repress gene expression corresponding to alternative ILC fates (23).

Several LDTFs involved in T lymphocyte development also control ILC development in mice, likely controlling this process by shaping their regulomes. These LDTFs include TCF-1 (encoded by the *Tcf7* gene) (76–78), TOX (79–81), Bcl11b (82–84), Runx (85, 86) and GATA-3 (87–91). During early T cell development in the thymus, TCF1 and Bcl11b sequentially switch T cell regulomes to a fate-committed configuration that possess lineage-specific accessible chromatin and nuclear organization (92, 93). Notably, in different lineages the same LDTFs can bind divergent sites in a context-dependent manner (85, 91, 94). Bcl11b, for example, targets different genomic locations in T cell progenitors and ILC2s, mediating lineage-specific gene regulation (94). Therefore, in depth experimental evaluation is required to map out complete ILC lineage- and state-specific transcriptional networks.

By contrast, some LDTFs bifurcate T and ILC development and contribute to initial steps in ILC regulome formation. NFIL3, for example, is essential for multiple stages of ILC lineage commitment and differentiation, but is dispensable in T cell development (95–98). High expression of NFIL3 in common ILC progenitors activates the NFIL3-TOX-TCF-1 cascade to permit differentiation of NK and ILC lineages from T cells and endorses NK and ILC lineage commitment (81, 96). NFIL3 is also required for the expression of ID2 (95, 99, 100); the latter is a key repressor that suppresses B and T cell fates to ensure ILC and NK cell specification (101–103). Depletion of ID2 enforces NK cells to acquire naïve T lymphocyte transcriptomic and epigenomic programs (102). Transient expression of PLZF (encoded at *Zbtb16*), another key LDTF associated with NKT cell development, plays an essential role in the commitment of ILC1, ILC2 and NCR^+^ ILC3 subsets and the exclusion of NK cell and LTi fates during early ILC development (104, 105). However, the potential of ILC precursors has been recently redefined by the generation of *Id2^RFP^Zbtb16^GFPcre^Bcl11b^tdTomato^* mice, showing that Id2^+^Zbtb16^+^ ILC precursors are able to give rise to NK cells, while Zbtb16 and Bcl11b control the late fates of ILC3 and ILC2 precursors (106).

In addition to LDTFs, signal-regulated transcription factors (SRTFs) activated by external signals can also lead to regulome transformation. In effector Th cells, activation-induced SRTFs (AP-1, IRF4 and BACH2) have a higher impact on the segregation of T cell populations than LDTFs do (T-bet, EOMES, ROR*γ*, and RORα) (73). Interestingly, the signaling pathways that dominate lymphocyte development and activation are in common at a significant level (39, 107). Polarization of distinct Th subsets requires activation of TCR-dependent SRTFs, including NF-κB, AP-1 and NFAT, as well as cytokine-mediated SRTFs like STATs and SMADs (108). Activation of STATs is essential for promoting differentiation of the Th lineages, as well as activation of ILCs and NK cells (109–112). The LDTFs T-bet and GATA-3 occupy lineage-specific REs in Th1 and Th2 cells, respectively; however, the absence of STAT4 and STAT6, which respectively shape Th1 and Th2 active enhancer landscapes, cannot be overcome by forced expression of LDTFs (56, 113). Additionally, polarization of Th17 cells relies on STAT3 and SMAD2/3 signaling pathways, which also promote activation of ILC3 and trans-differentiation of ILC1 or ILC2 lymphocytes to an ILC3-like phenotype (109, 114, 115). Other agonists, including cytokines and alarmins like IL-25, IL-33 and IL-18, along with leukotrienes, prostaglandin 2, and the neuropeptide neuromedin U can lead to NF-κB, AP-1 and NFAT activation (116–121).

**Figure 2:**
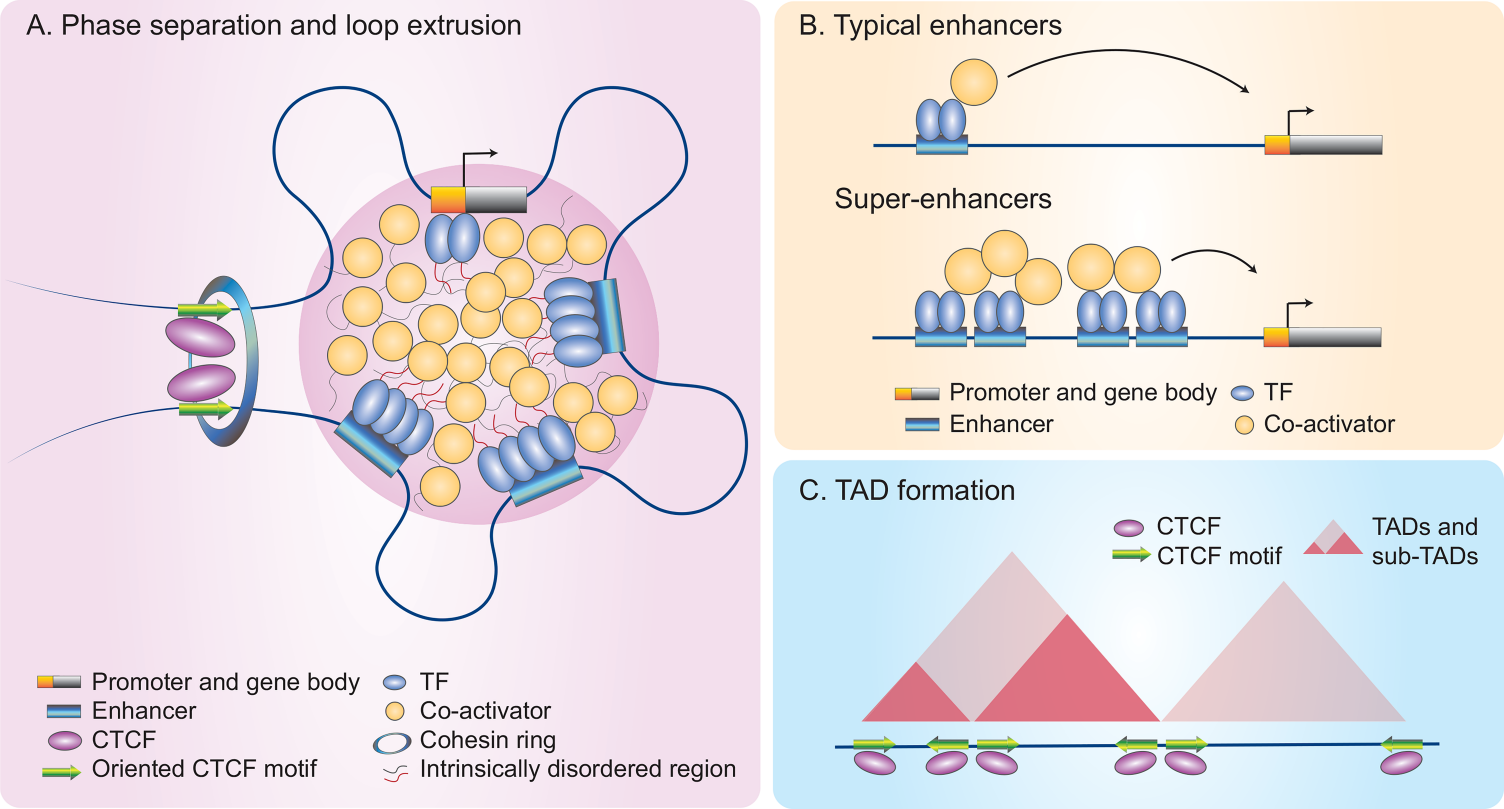
A model of rapid gene induction in NK cells through higher-order chromatin architecture and remodeling. Many inducible genes in NK cells are associated with super-enhancers (SEs) that can be orderly modulated by multi-dimensional epigenetic mechanisms (72). **(A)** Phase separation. Phase separation occurs as a dynamic process in which transcription factors (TFs) and co-activators form non-membrane bound condensates through weak multivalent protein-protein interactions of their intrinsically disordered region (130). Multi-loop hubs bring TF-bound regulatory elements (REs) and their target genes into close proximity to finetune gene expression. **(B)** Super-enhancers (SEs). SEs differ from typical enhancers as they recruit large numbers of TFs and transcriptional apparatus, including co-activators, to drive high magnitude of gene induction (130–132). **(C) **Topologically associating domains (TADs). Hi-C plots allow for visualization of three-dimensional TADs and sub-TADs, which form during the cohesin-mediated loop extrusion process. Looping can occur between two convergently oriented CCCTC-binding factor (CTCF) sites, using a cohesin ring that extrudes DNAs as shown in **(A)** (2, 133–135).

ILC regulomes are hard-wired to prime cytokines and other key effector genes for rapid responses. The paradigmatic view is that SRTFs facilitate rapid gene induction by activating enhancers primed during ILC development. For example, the SRTF STAT5 represents a central node in ILC development and acquisition of cell identity (122–124). However, rapid ILC activation relies on abilities of SRTFs to remodel *de novo* or latent enhancer landscapes for LDTF binding to their cognate DNA motifs in a sequence-specific manner (50, 125, 126). ILCs can further undergo chromatin remodeling in the context of infection or inflammation (127, 128), a process involving sequence-specific recognition of SRTFs (69, 72, 129). Interestingly, SRTF-activated *de novo* enhancer landscapes can further recruit LDTFs through a sequence-independent mechanism (72). Recent evidence indicates that TFs and co-activators with intrinsically disordered regions can form non-membrane bound condensates through weak multivalent protein-protein interactions, a dynamic process called phase separation (**Figure 2A**) (136–139). It remains to be determined whether the stimulation-dependent redistribution of LDTFs results from SRTF-mediated reorganization of phase separation, which contributes to biased loading of transcriptional machinery at super-enhancers (130–132).

## Super-Enhancers, a Reservoir of Transcriptional Machinery

Super-enhancers or stretch-enhancers (SEs), in contrast to typical enhancers, denote complex REs marked by high density deposition of transcription factors and enhancer marks (**Figure 2B**); these features are often indicative of key cell identity and disease-associated genes (57, 130, 140–145). The construction of SEs involves remodeling chromatin landscapes induced by both intrinsic and extrinsic signals to recruit large numbers of TFs and transcriptional apparatus that contains co-activators including Mediator (**Figures 2A, B**) (136, 140). Along with the formation of multi-loop hubs, the result is that regulatory elements and their target genes are brought into close proximity (130, 146).

Delineation of SEs in Th cells using the active enhancer-associated protein histone acetyltransferase p300, revealed that the majority of Th lineage- and function-defining genes, including cytokines (*Ifng, Il4, Il10, Il17a* and *Il17f*) and key transcription factors (*Tbx21*, *Gata3*, *Rorc* and *Rora*), form SE structures during Th differentiation (57). These findings are consistent with multiple REs or locus control regions previously identified to be in close proximity to cytokine genes, including *Ifng*, Th2 cytokines (*Il4-Il13-Il5*) and the *Il17a-Il17f* locus (67, 147). Profiling SEs in human tonsillar ILCs and T cells by active enhancer mark histone 3 lysine 27 acetylation (H3K27Ac) clearly differentiates ILCs from Th cells (63).

Recent studies revealed that SE structures also are indicative of a high magnitude of gene induction. Within hours of cytokine stimulation, SRTFs such as NF-κB and STATs rapidly establish SEs at effector gene loci in innate immune cells (macrophages and NK cells) to quickly provoke pro-inflammatory transcriptomes (72, 148–150). This process involves the recruitment of p300 to catalyze H3K27Ac histone modification to both primed and *de novo* enhancers for prompt gene induction. In activated NK cells, *de novo* SEs are linked to highly-inducible genes, suggesting the rapid construction of SE structures boosts the magnitude of immediate transcriptional activity (**Figure 2**) (72).

## Solution for Physical Distancing—Nuclear Compartmentalization and Higher-Order Chromatin Architecture

Within the nucleus, the stretch of one-meter long DNA is segregated into active (euchromatin) and inactive (heterochromatin) territories, which are spatially organized into individual regulatory domains, designated topologically associating domains (TADs) (5, 151, 152). TADs are formed *via* an extrusion process mediated by a cohesin ring and blocked by two convergently orientated CCCTC-binding factor (CTCF) sites (**Figures 2A, C**) (2, 133, 134, 153–158). CTCF is a chromatin organizer that dominates higher-order chromatin architecture and a multifunctional zinc finger TF that functions as an activator, a repressor or an insulator depending on co-localized molecules and how the chromatins are looped (135, 159).

Although most TADs are largely invariant across cell types, TADs and nested sub-TADs may also be cell-type specific, and thus underlie cell identity and discrete functions (151, 160). Even though CTCF is ubiquitously expressed and constitutively occupies TAD boundaries across different cell types (135), dynamic enhancer-promoter interactions and selective CTCF deposition at cell type-specific genes does occur. The CTCF-cohesin-mediated 3D chromatin architecture dominates many biological processes including regulation of key cytokines. Global CTCF deficiency leads to impaired IFN-γ and Th2 cytokine production in Th1 and Th2 cells, respectively (161, 162).

Other TFs also actively control chromatin topology. In Th1 cells, T-bet coordinates with CTCF to regulate *Ifng* locus 3D structure and full expression capacity (161). Crystal structure studies indicate that the T-bet DNA binding domain forms a dimer that allows T-bet to bind two independent DNA motifs distal from each other, suggesting the role of T-bet in loop formation (163). IL-2-mediated STAT5 activation also reconstruct T cell regulomes by remodeling SE landscapes and 3D regulatory domains that facilitate induction of IL-2 target genes (164). Dissecting the specific and dynamic roles of LDTFs and SDTFs in higher-order chromatin architecture in resting and activated ILCs will have important implications for understanding ILC gene regulation in health and disease.

## Non-Coding RNAs in Lymphocyte Regulation

Short non-coding RNAs, including microRNAs (miRNAs), as well as long non-coding RNAs (lncRNAs), including circular RNAs (circRNAs), are key players in post-transcriptional regulation and chromatin remodeling in innate and adaptive lymphocytes (165, 166). Mechanistically, lineage-specific miRNAs and lncRNAs are linked to super-enhancers and can control target genes *in cis* or *in trans* (167–170). For example, miR-29 directly regulates IFN-γ production in NK, CD4^+^ and CD8^+^ T cells by targeting IFN-γ mRNA or indirectly *via* suppression of LDTFs EOMES and T-bet (171, 172). Other miRNAs including miR-155 and miR-17~92 promote Th1 immunity (173–176). Interestingly, miR-17~92 also promotes Th2 immunity in asthma affected airways (177), pointing to complex, less well-understood functions. In ILC1s, miR-142 plays a central role in IL-15-mediated NK cell survival, trafficking, homeostasis and defense against viral infection (178). Deficiency of miR-142 led to aberrant ILC1-like cell accumulation, potentially driven by TGF-β.

lncRNAs are critical for CD8^+^ (179, 180) and CD4^+^ T cell differentiation (181, 182). The *Ifng* locus itself is positively regulated by the lncRNA *Ifng-as1* (also known as NeST or Tmevpg1) as a mechanism to enhance *Ifng* expression in Th1 cells (183–186). The expression of *Ifng-as1* is dependent on remodeling of the proximal and distal enhancers by T-bet, recruiting TFs NF-κB and Ets1 to drive *Ifng-as1* transcription (187). *Ifng-as1* is capable of engaging the chromatin modifying enzyme WDR5 that alters histone 3 methylation at the *Ifng* locus (184). Deletion of *Ifng-as1*, within the *Ifng* extended locus, led to disruption of chromatin organization and reduced *Ifng* expression, indicating its role in maintenance of the chromatin architecture of the *Ifng* extended locus. This was in part due to the deletion of a critical CTCF site that acted as a functional insulator (183).

lncRNAs can also modulate ILC development and function. For instance, the ILC1-specific lncRNA *Rroid* promotes the expression of *Id2*, a transcription regulator that represses adaptive lymphocyte cell fate, and is essential for ILC1 development (188). The lncRNA *lncKdm2b* is highly expressed in ILC3s and plays a key role in ILC3 maintenance through activation of the TF Zfp292 (189). On the other hand, the circRNA *circKcnt2* inhibits *Batf* expression, which results in inhibition of ILC3 activation and IL-17 expression (190). Exactly how these IncRNAs precisely exert their effects and whether these mechanisms are conserved between innate and adaptive lymphocytes, however, remains unclear.

## Concluding Remarks

Regulation of key cell identity and cytokine genes in lymphocytes requires carefully orchestrated epigenetic mechanisms and remodeling of the chromatin landscape by transcription factors (LDTFs and SRTFs), super-enhancers, TAD formation, CTCF-anchored loops and non-coding RNAs. Exploration of these avenues in both local tissue and systemic environments holds promise in furthering our understanding of ILC and T cell regulomes. Several fundamental questions remain: how are nuclear compartmentalization and phase separation altered during lymphocyte development and activation? How do LDTFs and other co-activators developmentally shape and maintain immune cell regulomes? How do divergent chromatin landscapes respond to distinct pathogen invasion? What are the roles of SRTFs in the redistribution of transcriptional apparatus to mount an adequate immune response? How do super-enhancers coordinate different TFs and co-activators in the 3D space to direct final transcriptional output?

The rapid improvement in genome-wide epigenomic and single-cell transcriptomic profiling has provided a new angle to view global chromatin landscapes and transcriptional networks, even in rare populations such as ILCs. However, we are still yet to fully understand how novel key factors (DNAs, RNAs and proteins) asymmetrically distribute in the nuclei and physically interplay with each other in a context-dependent manner. The potential of newly developed techniques in the fields of molecular biology, fixed-cell microscopy, live-cell imaging, cryo-EM and genome editing may help to further our understanding. We are rapidly emerging into an era of epigenomic research that will allow us to decipher the mechanisms for lineage commitment and cytokine regulation in detail. Ultimately, we seek to identify key factors, signaling pathways or epigenetic modulations that can be targeted to prevent and/or control lymphocyte-mediated inflammation in diseases.

## Author Contributions:

H-YS conceived and wrote the first draft of the manuscript. NF and GS wrote sections of the manuscript and drafted the figures. JJO, NF, and GS reviewed and revised the text and figures. All authors contributed to the article and approved the submitted version.

## Funding

This review is supported by the NEI Intramural Research Program fund (ZIAEY000569-01).

## Conflict of Interest

The authors declare that the research was conducted in the absence of any commercial or financial relationships that could be construed as a potential conflict of interest.

## Abbreviations

3C, chromatin conformation capture; ATAC-seq, Assay for Transposase-Accessible Chromatin using sequencing; ChIP, chromatin immunoprecipitation; circRNA, circular RNA; CTCF, CCCTC-binding factor; CTL, cytotoxic T lymphocyte; EOMES, eomesodermin; GATA-3, GATA-binding protein 3; H3K27Ac, histone 3 lysine 27 acetylation; IFN-γ, interferon gamma; IL, interleukin; ILC, innate lymphoid cell; LDTF, Lineage-Determining Transcription Factor; lncRNA, long non-coding RNA; LTi, Lymphoid tissue inducer cell; miRNA, microRNA; NCR, natural cytotoxicity receptor; NF-κB, Nuclear factor-κB; NK, natural killer cell; NKT, natural killer T cell; RE, regulatory element; RORγt, RAR-related orphan receptor gamma; SE, super-enhancer; SRTF, Signal-Regulated Transcription Factor; STAT, signal transducer and activator of transcription; TAD, topologically associating domains; TCR, T cell receptor; TF, transcription factor; Th cell, helper T cell.

## Glossary

Regulatory elements (REs): Non-coding sequences that control gene expression through physical interactions and recruitment of transcription factors and transcriptional apparatus. REs can be functionally classified as promoters, enhancers, silencers and insulators depending on their genomic locations and bound molecules.
Regulomes: The whole set of regulatory components that control cell identity and functionality. These components include transcription factors and their co-activators/co-repressors that modulate gene expression levels through chromatin remodeling.
Innate lymphoid cells (ILCs): Innate immune cells that execute effector functions as T lymphocytes without the expression of T cell receptors or the need of T cell receptor signaling for activation. ILCs are enriched at barrier surfaces and play critical roles in tissue integrity, homeostasis and the primary immune response.
Pioneer transcription factors (TFs): TFs that bind specific DNA sequences among compacted chromatin regions that are tightly wrapped by nucleosomes. Pioneer TFs then initiate stepwise chromatin remodeling to “open” the chromatin and recruit other non-pioneer TFs for gene regulation.
Lineage-determining transcription factors (LDTFs): Also called master regulators. Transcription factors that are expressed at specific developmental stages for cell fate decisions and lineage commitment. LDTFs are often recognized as pioneer TFs.
Signal-regulated transcription factors (SRTFs): Transcription factors that are activated by external stimuli and bind to REs to convert environmental inputs to transcriptional outputs.
Poised enhancers: Enhancers that have been recognized and opened by pioneer TFs (often LDTFs) during development and can be further activated by SRTFs upon stimulation.
De novo enhancers: Closed, inactive enhancer loci that can be rewired by SRTFs upon stimulation.
Phase separation: A physicochemical process by which molecules segregate into a dense phase and a dilute phase. Recent studies revealed that transcription factors and co-factors can utilize the interaction of their intrinsically disordered regions to induce phase separation and form biomolecular condensates for enhancer complex assembly.
Topologically associating domains (TADs): Megabase-sized genomic loci in proximity that form an interacting chromatin hub in three-dimensional nuclear space. The boundaries of TADs often define enhancer targets and the genes that are co-regulated.
